# Supplementary material for: Compression pressure of the external jugular vein for the assessment of intravascular volume status in decompensated cirrhosis: A pilot study
Source: JHEP Rep. 2025 Dec 13;8(3):101712. doi: 10.1016/j.jhepr.2025.101712 (PMC12907074; doi:10.1016/j.jhepr.2025.101712)
Supplement: Multimedia component 1 [file mmc1.pdf]

Compression pressure of the external jugular vein for the assessment of intravascular volume status in decompensated cirrhosis: A pilot study

Daniel Segna, Benjamin Messerli, Ulrich Baumann, Jaume Bosch, Annalisa Berzigotti

Table of content

Supplementary materials and methods……………………………………………….2

Supplementary materials and methods

Measurement details

All POCUS and CPMX2 measurements were performed at following time points: 1) immediately before PLR, 2) during sustained PLR (IVC-POCUS at 1 minute, repeated EJV compression pressures by CPMX2 during 2 minutes), 3) immediately before IV albumin infusion and any intervention (i.e. therapeutic paracentesis), 4) within 2 hours after last IV albumin infusion.

Measurement details for EJV-CPMX2

Patients were examined in a supine position. The probe of the CPMX2 System was applied with increasing pressure on the EJV by the investigator up to the moment in which the vessel collapsed. The ultrasound views and the corresponding extrinsically applied pressures were transferred in real time to a tablet as part of CPMX2. This allows the investigator to detect the occlusion pressure (measured in mmHg) and to start decompressing the vessel. Measurements were recorded and stored as ultrasound movie clips on the tablet for external review of the accurate occlusion pressure. Measurements were repeated at least 3 times in a stable patient position before PLR, as well as before and after IV albumin. During PLR, we performed repetitive measurements for at least 7 times within 2 minutes of sustained maneuver.

Measurement details for IVC-POCUS

Two independent experienced sonographers measured IVC parameters with a convex probe on two different devices (Philips Affiniti 50G – ultrasound system and GE Healthcare VenueFitTM), and images were saved in a centrally coded register for internal review. Maximum and minimum IVC diameters were measured on spontaneous breathing without a sniff. IVCCI was calculated according the formula: $IVCCI = (IVC^{max} - IVC^{min})/IVC^{max}$. Due to moderate and large volume ascites and/or reduced visibility due to bloating, obesity or air interposition, we measured IVC parameters in a mid-axillary window 1-2 cm distally to the hepatic vein inlet into the IVC [1].

Supplementary reference

1. Kaptein MJ, Kaptein EM. Inferior Vena Cava Collapsibility Index: Clinical Validation and Application for Assessment of Relative Intravascular Volume. Adv Chronic Kidney Dis. 2021;28(3):218-26.
