# Supplementary material for: Compression pressure of the external jugular vein for the assessment of intravascular volume status in decompensated cirrhosis: A pilot study
Source: JHEP Rep. 2025 Dec 13;8(3):101712. doi: 10.1016/j.jhepr.2025.101712 (PMC12907074; doi:10.1016/j.jhepr.2025.101712)
Supplement: Multimedia component 4 [file mmc4.pdf]

JHEP|Reports

Compression pressure of the external jugular vein for the assessment of intravascular volume status in decompensated cirrhosis: A pilot study

Authors

Daniel Segna, Benjamin Messerli, Ulrich Baumann, Jaume Bosch, Annalisa Berzigotti

Correspondence

daniel.segna@extern.insel.ch (D. Segna).

Graphical abstract

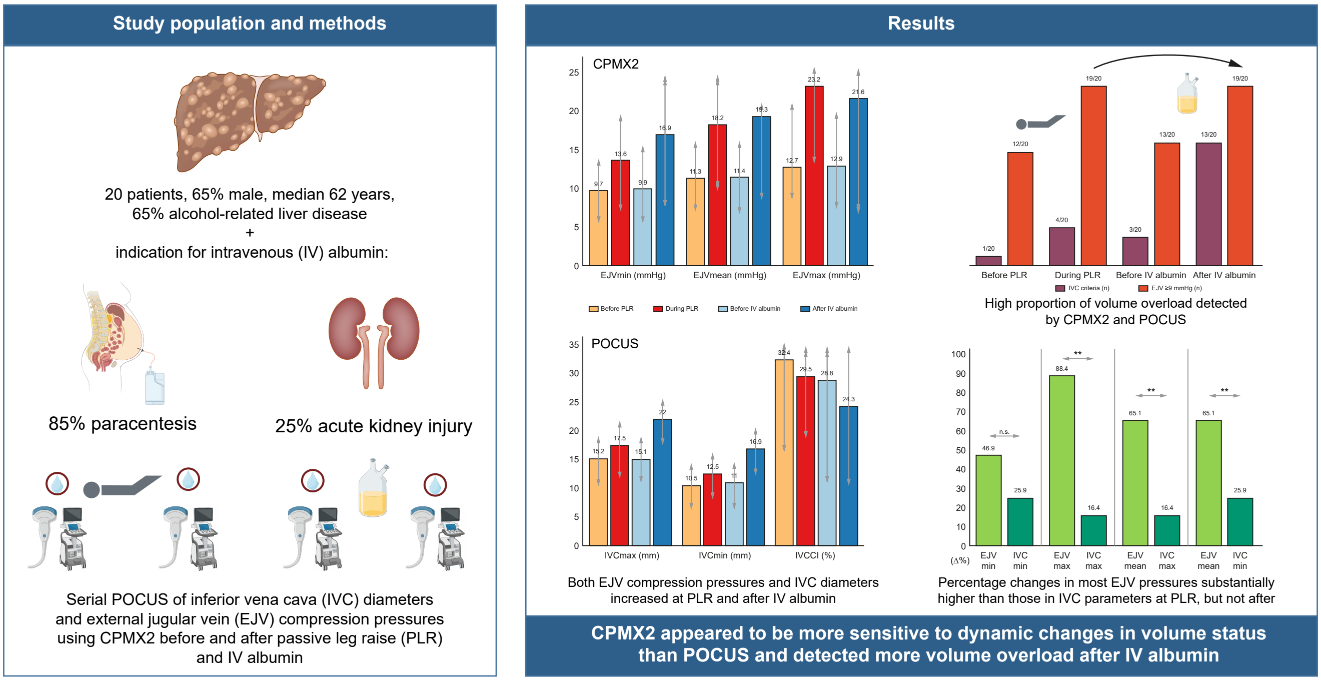

Highlights:

- POCUS and CPMX2 detected significant intravascular volume changes during PLR and after IV albumin.
- Both CPMX2 and POCUS identified a high frequency of potential post-albumin volume overload.
- CPMX2 was more sensitive than POCUS for detecting dynamic volume shifts during PLR.
- CPMX2 may enable individualized fluid management in decompensated cirrhosis after further validation.

Impact and implications:

Intravascular volume overload after albumin infusion is a major concern in patients with decompensated cirrhosis and was observed in its most severe form in 20% of patients using POCUS-IVC (point-of-care ultrasound of the inferior vena cava). In this pilot study, we detected substantial volume shifts during passive leg raise and after albumin infusion using both POCUS-IVC and a novel external jugular vein compression technique (CPMX2) in parallel. CPMX2 appeared more sensitive to dynamic changes during passive leg raise and identified a larger proportion of patients with intravascular volume overload following intravenous albumin. These findings suggest that CPMX2 could serve as a promising non-invasive tool for early assessment and individualized monitoring of volume status in decompensated cirrhosis.

https://doi.org/10.1016/j.jhepr.2025.101712

© 2025 The Author(s). Published by Elsevier B.V. on behalf of European Association for the Study of the Liver (EASL). This is an open access article under the CC BY license (http://creativecommons.org/licenses/by/4.0/).

JHEP|Reports

Compression pressure of the external jugular vein for the assessment of intravascular volume status in decompensated cirrhosis: A pilot study

Daniel Segna[1,*], Benjamin Messerli[1], Ulrich Baumann[2], Jaume Bosch[1,3], Annalisa Berzigotti[1,3]

JHEP Reports **2026**. vol. 8 | 1–4

Check for updates

Background & Aims: Patients with decompensated cirrhosis are susceptible to iatrogenic hypervolemia. A pilot study using point-of-care ultrasound (POCUS) of the inferior vena cava (IVC) found severe hypervolemia in 20% of patients after intravenous (IV) albumin, despite adherence to current guidelines. CPMX2 is a novel non-invasive device that uses external jugular vein (EJV) compression to assess central venous pressure, but its ability to predict post-albumin hypervolemia has not been studied. We aimed to track changes in intravascular volume status during passive leg raise (PLR) and IV albumin using both methods.

Methods: IVC diameters (IVCmin, IVCmax) and IVC collapsibility index (IVCCI) were measured by POCUS, and EJV compression pressures (EJVmin, EJVmax, EJVmean) were measured by CPMX2, in parallel before and during PLR, as well as before and after IV albumin. Potential intravascular overload was defined as IVCmax >2.1 cm and IVCCI <50%, or EJVmean ≥9 mmHg.

Results: In this prospective cohort of 20 patients (35% women; median age 62 years; mean BMI 25.7 kg/m^2; 55% Child-Pugh B; 80% receiving paracentesis), all IVC diameters and EJV compression pressures increased during PLR and after IV albumin. Percentage changes in EJV compression pressures were greater than changes in IVC diameters during PLR (mean EJVmax +88%, EJVmean +65% *vs.* IVCmax +16%, IVCmin +26%; all p <0.01). After IV albumin, changes were comparable between the methods (mean EJVmax +68%, EJVmin +80%, EJVmean +75% vs IVCmax +58%, IVCmin +79%). Potential post-albumin volume overload occurred in most cases (POCUS 65%, CPMX2 95%).

Conclusions: Both POCUS and CPMX2 detected substantial volume changes during PLR and following IV albumin. CPMX2 appeared more sensitive to dynamic changes during PLR and identified more patients with potential post-albumin volume overload, suggesting it may help individualize fluid management in decompensated cirrhosis.

© 2025 The Author(s). Published by Elsevier B.V. on behalf of European Association for the Study of the Liver (EASL). This is an open access article under the CC BY license (http://creativecommons.org/licenses/by/4.0/).

Introduction

Patients with decompensated cirrhosis are at risk for iatrogenic volume overload,[1–3] a significant complication and clinical challenge following intravenous (IV) albumin infusion. IV albumin is commonly used to manage complications of portal hypertension, including large-volume ascites requiring paracentesis, acute kidney injury (AKI) with or without hepatorenal syndrome, and spontaneous bacterial peritonitis. In a prospective cohort of 55 patients and 81 measurements using point-of-care ultrasound (POCUS) of the inferior vena cava (IVC) diameters and collapsibility,[4] we identified intravascular overload in nearly half of patients, and severe intravascular volume overload in approximately 20% immediately after IV albumin, even with strict adherence to current albumin dosage recommendations.[5,6] Severe volume overload correlated with elevations in NT-proBNP (N-terminal pro-brain natriuretic peptide), suggesting potential clinical relevance. Interestingly, traditional tests for assessing fluid responsiveness, such as passive leg raise (PLR),[7] which are validated in intensive care settings, failed to predict impending iatrogenic overload post-infusion as assessed by IVC-POCUS. This may be attributable to variable increases in intra-abdominal pressure caused by large-volume ascites, which can obscure accurate assessment of intravascular volume shifts. Consequently, there is an unmet need for reliable, non-invasive tools to monitor fluid responsiveness and predict volume overload in decompensated cirrhosis. CPMX2 (*Compremium AG, Switzerland*) is an innovative non-invasive medical device designed to assess central venous pressure by applying controlled compression to the external jugular vein (EJV),[8] and has been validated in critically ill patients requiring intensive care, demonstrating promising accuracy, safety and applicability.[9,10] However, its predictive accuracy in patients with decompensated cirrhosis, particularly in the context of post-albumin infusion, remains unexplored.

* Corresponding author. Address: Department for Visceral Surgery and Medicine, Bern University Hospital, University of Bern, Freiburgstrasse 21, 3010 Bern, Switzerland. Tel.: +41 31 63 2 02 61.
E-mail address: daniel.segna@extern.insel.ch (D. Segna).
https://doi.org/10.1016/j.jhepr.2025.101712

Patients and methods

In this prospective single-center pilot study comparing POCUS of the IVC and the novel compression pressure technique CPMX2, we aimed to track changes in intravascular volume status during PLR and following IV albumin administration. We collected all data in an observational setting during standard medical treatment from July 2024 and April 2025 at the Department of Visceral Surgery and Medicine, Bern University Hospital, Bern, Switzerland. The study protocol had been approved by the local institutional board (BASEC 2024-D0007).

For this pilot project, we included ambulatory and hospitalized adult patients with decompensated cirrhosis and an indication for IV albumin therapy according to current international guidelines (such as therapeutic paracentesis, AKI, AKI-hepatorenal syndrome, spontaneous bacterial peritonitis)[5,6] upon informed consent. Cirrhosis was diagnosed following combined histological, biochemical, radiologic and/or clinical information. Patients were excluded if they had a history of liver transplantation, right heart failure, clinical evidence of pulmonary edema, recent hemodynamic shock, current pregnancy, unavailable transthoracic echocardiography within 3 months of study inclusion, admission to intensive or intermediate care at baseline, recent IV albumin within the past 5 days, or contraindications to PLR (*e.g.* increased intracranial pressure) or IV albumin (*e.g.* anaphylaxis). Our primary outcomes included absolute and percentage changes in external jugular vein (EJV) occlusion pressures – maximum (EJVmax), minimum (EJVmin), and mean (EJVmean) – as well as inferior vena cava (IVC) diameters –maximum (IVCmax) and minimum (IVCmin) – and collapsibility (IVCCI) measured before and during PLR, and before and after IV albumin (see supplementary materials and methods for measurement details). As secondary outcomes, we assessed potential intravascular volume overload, defined as IVCmax >2.1 cm and IVCCI <50%, using POCUS, and EJVmean ≥9 mmHg for CPMX2. We implemented a rather conservative EJV threshold to increase sensitivity for detecting intravascular volume overload, considering the highly heterogeneous literature on the limit of increased central venous pressure.[11]

Results

In our prospective cohort of 20 patients with decompensated cirrhosis (35% women, median age 62 years, mean BMI 25.7 kg/m^2, 55% Child-Pugh B, median MELD 15, 80% paracentesis as an indication for IV albumin, Table 1), all IVC diameters and EJV compression pressures considerably increased during PLR and after IV albumin infusion (Fig. 1A,B). Percentage changes in most EJV compression pressures were markedly higher than those observed in IVC diameters during PLR (mean EJVmax +88.4%, EJVmean +65.1% *vs.* IVCmax +16.4%, IVCmin +25.9%; p <0.01, Fig. 1C,D). On the other hand, percentage changes in the parameters obtained by the two techniques were comparable after IV albumin infusion (mean EJVmax +68.3%/EJVmin +80.3%, EJVmean +74.7% *vs*. IVCmax +57.5%/IVCmin +78.9%, Fig. 1C,D). Intravascular volume overload after IV albumin was very frequent, occurring in the majority of observed cases (POCUS: 65.0%, CPMX2: 95.0%, Fig. 1E). CPMX2 showed a substantially higher agreement for volume overload at PLR and after IV albumin than POCUS (90.0% *vs.* 23.1%). There was a weak positive correlation between percentage changes in EJVmin post-infusion and previously infused albumin dosages (rho 0.486, p = 0.03), while absolute values or dynamics in other IVC and EJV parameters showed no clear correlation with the amount of albumin infused.

Table 1. Baseline characteristics of the included population.

**Sex**	
Male (n, %)	13 (65%)
Female (n, %)	7 (35%)
**Age** (years, median, IQR)	62 (55.3-66.8)
**BMI** (kg/m^2, median, IQR)	24.9 (22.0-29.7)
**Child-Pugh score**	
B (n, %)	11 (55%)
C (n, %)	9 (45%)
**MELD score** (median, IQR)	18 (11-25)
**Etiology of cirrhosis**	
ALD (n, %)	13 (65%)
MetALD (n, %)	1 (5%)
MASH (n, %)	1 (5%)
Miscellaneous (n, %)	5 (25%)
**Indication for IV albumin infusion**	
Large volume paracentesis (n, %)	17 (85%)
Acute kidney injury (n, %)	5 (25%)
Spontaneous bacterial peritonitis	1 (5%)
**Dosage for albumin infusion** (g, median, IQR)	40 (20-40)
**Quantity of ascites removed** (L, median, IQR)	5.5 (3.3-7.3)

ALD, alcohol-related liver disease; IV, intravenous; MASH, metabolic-dysfunction associated steatohepatitis; MELD, model for end-stage liver disease; MetALD, metabolic dysfunction and alcohol-related liver disease.

Discussion

In this pilot project, we found that both POCUS-based IVC assessment and CPMX2-derived EJV compression pressures detect considerable intravascular volume changes during PLR and following IV albumin infusion in patients with decompensated cirrhosis. CPMX2 appeared more sensitive to dynamic changes during PLR and identified a greater proportion of patients with intravascular volume overload after IV albumin. These findings suggest that CPMX2 could serve as a promising, non-invasive tool for early characterization of volume status and fluid responsiveness, which could help tailor albumin therapy, particularly in the context of large volume paracentesis. This may represent an advantage over IVC-POCUS, which can yield false negatives due to elevated intra-abdominal pressure. Nevertheless, an important limitation of our study is the absence of a direct comparison between CPMX2, POCUS, and invasive measurements of central venous pressure (the current reference standard), which is required to verify the diagnostic accuracy of both non-invasive techniques in detecting volume overload. Furthermore, associations between detected changes in intravascular volume status and cardiopulmonary and liver-related events deserve further investigation. Future studies should compare CPMX2 and POCUS with invasive measurements, and evaluate CPMX2-guided albumin dosing as part of a personalized therapeutic approach in decompensated cirrhosis.

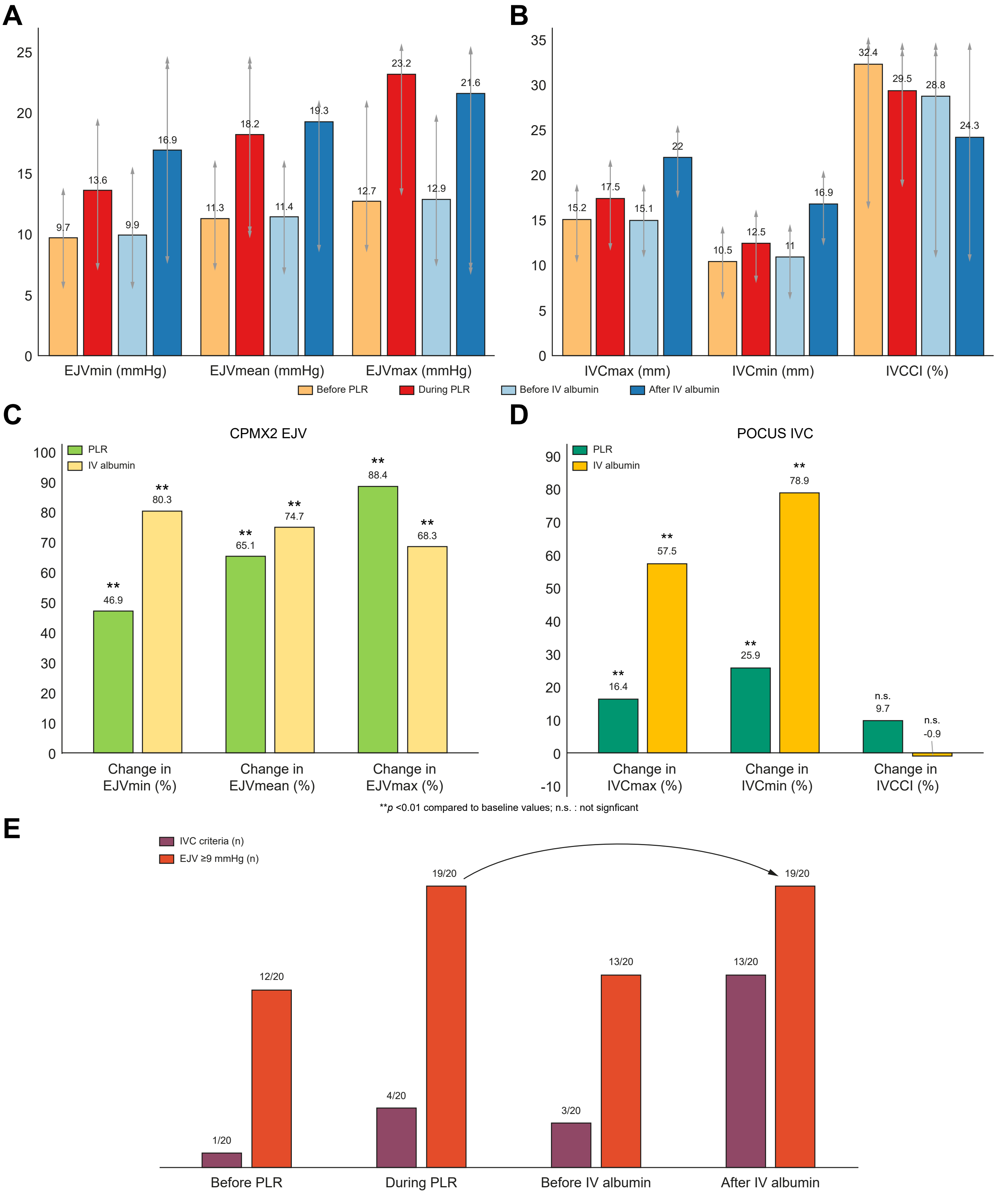

Fig. 1. EJV compression pressures and IVC diameters and occurence of any potential volume overload during passive leg raise and albumin infusion . (A) EJV occlusion pressures (mean, SD) before/after PLR and IV albumin. (B) IVC diameters and collapsibility before/after PLR and IV albumin. (C) Percentage changes in EJV occlusion pressures during PLR and after IV albumin. (D) Percentage changes in IVC parameters during PLR and after IV albumin. (E) Occurrence of any volume overload defined by IVC and EJV criteria. Percentage changes in EJV occlusion pressures and IVC parameters after PLR and albumin infusion were analyzed using paired Wilcoxon signed-rank tests or one-sample t-tests, as appropriate. EJV, external jugular vein; IV, intravenous; IVC, inferior vena cava; IVCCI, IVC collapsibility index; IVCmax, maximum IVC diameter; IVCmin, minimum IVC diameter; PLR, passive leg raise.

Affiliations

[1]*Department of Visceral Surgery and Medicine, Inselspital, Bern University Hospital, University of Bern, Switzerland;* [2]*Compremium AG, Bern, Switzerland;* [3]*Department for BioMedical Research, Visceral Surgery and Medicine, University of Bern, Switzerland*

Abbreviations

AKI, acute kidney injury; IV, intravenous; IVC, inferior vena cava; IVCCI, IVC collapsibility index; IVCmax, maximal diameter of the inferior vena cava during an entire respiratory cycle; IVCmin, minimal diameter of the inferior vena cava during an entire respiratory cycle; PLR, passive leg raise; POCUS, point-of-care ultrasound.

Financial support

The work has been supported by Compremium AG, Switzerland. JB was supported by the Swiss Liver Foundation.

Conflicts of interest

DS reports travelling fees from Gilead and Falk. AB is a consultant for Boehringer-Ingelheim and Astra Zeneca; Speakers' fees from GE Healthcare and Hologic. JB was a consultant for AstraZeneca, NovoNordisk, Boehringer Ingelheim and Resolution Therapeutics. BM has nothing to disclose. UB is chief medical officer and founder of Compremium AG.

Please refer to the accompanying ICMJE disclosure forms for further details.

Authors' contributions

Study design: DS, AB, JB, UB. Study set-up: DS, AB, JB, UB; Data collection and analysis: DS, BM; Manuscript writing: DS, AB, JB, BM, UB. Manuscript revision; DS, AB, BM, UB. All authors approved the current version of the manuscript.

Data availability

All datasets generated and/or analyzed during this study can be obtained from the corresponding author upon reasonable request.

Acknowledgements

We would like to thank Lilian Smith and Judith Freiburghaus for their support during the whole duration of the project.

Supplementary data

Supplementary data to this article can be found online at https://doi.org/10.1016/j.jhepr.2025.101712.

References

[1] Velez JCQ, Petkovich B, Karakala N, et al. Point-of-Care echocardiography Unveils misclassification of acute kidney injury as hepatorenal syndrome. Am J Nephrol 2019;50(3):204–211. Epub 20190808.

[2] Caraceni P, Riggio O, Angeli P, et al. Long-term albumin administration in decompensated cirrhosis (ANSWER): an open-label randomised trial. Lancet 2018;391(10138):2417–2429. Epub 20180601.

[3] China L, Freemantle N, Forrest E, et al. A randomized trial of albumin infusions in hospitalized patients with cirrhosis. N Engl J Med 2021;384(9):808–817.

[4] Segna D, Brazerol F, Radu P, et al. Point-of-care ultrasound of the inferior vena cava for intravascular volume assessment during intravenous albumin infusion in patients with cirrhosis. JHEP Rep 2025:101559.

[5] de Franchis R, Bosch J, Garcia-Tsao G, et al. Baveno VII - renewing consensus in portal hypertension. J Hepatol 2022;76(4):959–974. Epub 20211230.

[6] European Association for the Study of the Liver. EASL Clinical Practice Guidelines for the management of patients with decompensated cirrhosis. J Hepatol 2018;69(2):406–460. Epub 20180410.

[7] Monnet X, Marik P, Teboul JL. Passive leg raising for predicting fluid responsiveness: a systematic review and meta-analysis. Intensive Care Med 2016;42(12):1935–1947. Epub 20160129.

[8] Baumann UA, Marquis C, Stoupis C, et al. Estimation of central venous pressure by ultrasound. Resuscitation 2005;64(2):193–199.

[9] Thalhammer C, Siegemund M, Aschwanden M, et al. Non-invasive central venous pressure measurement by compression ultrasound–a step into real life. Resuscitation 2009;80(10):1130–1136. Epub 20090725.

[10] Uthoff H, Siegemund M, Aschwanden M, et al. Prospective comparison of noninvasive, bedside ultrasound methods for assessing central venous pressure. Ultraschall Med 2012;33(7). E256-e62. Epub 20120601.

[11] Joseph A, Petit M, Vignon P, et al. Fluid responsiveness and venous congestion: unraveling the nuances of fluid status. Crit Care 2024;28(1):140.

Keywords: cirrhosis; point of care ultrasound; volume management; inferior vena cava; external jugular vein; compression.

Received 15 September 2025; received in revised form 4 December 2025; accepted 9 December 2025; Available online 13 December 2025

Journal of Hepatology, Volume *8*

Supplemental information

Compression pressure of the external jugular vein for the assessment of intravascular volume status in decompensated cirrhosis: A pilot study

Daniel Segna, Benjamin Messerli, Ulrich Baumann, Jaume Bosch, and Annalisa Berzigotti

Compression pressure of the external jugular vein for the assessment of intravascular volume status in decompensated cirrhosis: A pilot study

Daniel Segna, Benjamin Messerli, Ulrich Baumann, Jaume Bosch, Annalisa Berzigotti

Table of content

Supplementary materials and methods……………………………………………………2

Supplementary materials and methods

Measurement details

All POCUS and CPMX2 measurements were performed at following time points: 1) immediately before PLR, 2) during sustained PLR (IVC-POCUS at 1 minute, repeated EJV compression pressures by CPMX2 during 2 minutes), 3) immediately before IV albumin infusion and any intervention (i.e. therapeutic paracentesis), 4) within 2 hours after last IV albumin infusion.

Measurement details for EJV-CPMX2

Patients were examined in a supine position. The probe of the CPMX2 System was applied with increasing pressure on the EJV by the investigator up to the moment in which the vessel collapsed. The ultrasound views and the corresponding extrinsically applied pressures were transferred in real time to a tablet as part of CPMX2. This allows the investigator to detect the occlusion pressure (measured in mmHg) and to start decompressing the vessel. Measurements were recorded and stored as ultrasound movie clips on the tablet for external review of the accurate occlusion pressure. Measurements were repeated at least 3 times in a stable patient position before PLR, as well as before and after IV albumin. During PLR, we performed repetitive measurements for at least 7 times within 2 minutes of sustained maneuver.

Measurement details for IVC-POCUS

Two independent experienced sonographers measured IVC parameters with a convex probe on two different devices (Philips Affiniti 50G – ultrasound system and GE Healthcare VenueFitTM), and images were saved in a centrally coded register for internal review. Maximum and minimum IVC diameters were measured on spontaneous breathing without a sniff. IVCCI was calculated according the formula: $IVCCI = (IVC^{max} - IVC^{min})/IVC^{max}$. Due to moderate and large volume ascites and/or reduced visibility due to bloating, obesity or air interposition, we measured IVC parameters in a mid-axillary window 1-2 cm distally to the hepatic vein inlet into the IVC [1].

Supplementary reference

1. Kaptein MJ, Kaptein EM. Inferior Vena Cava Collapsibility Index: Clinical Validation and Application for Assessment of Relative Intravascular Volume. Adv Chronic Kidney Dis. 2021;28(3):218-26.
